# Nuclear Envelope Remnants: Fluid Membranes Enriched in STEROLS and Polyphosphoinositides

**DOI:** 10.1371/journal.pone.0004255

**Published:** 2009-01-23

**Authors:** Marie Garnier-Lhomme, Richard D. Byrne, Tina M. C. Hobday, Stephen Gschmeissner, Rudiger Woscholski, Dominic L. Poccia, Erick J. Dufourc, Banafshé Larijani

**Affiliations:** 1 Cell Biophysics Laboratory, Lincoln's Inn Fields Laboratories, Cancer Research UK, London, United Kingdom; 2 Electron Microscopy Unit, Lincoln's Inn Fields Laboratories, Cancer Research UK, London, United Kingdom; 3 Division of Cell and Molecular Biology, Imperial College London, London, United Kingdom; 4 Department of Biology, Amherst College, Amherst, Massachusetts, United States of America; 5 UMR 5248 CNRS-Université Bordeaux 1-ENITAB, IECB, Pessac, France; University of California, Berkeley, United States of America

## Abstract

**Background:**

The cytoplasm of eukaryotic cells is a highly dynamic compartment where membranes readily undergo fission and fusion to reorganize the cytoplasmic architecture, and to import, export and transport various cargos within the cell. The double membrane of the nuclear envelope that surrounds the nucleus, segregates the chromosomes from cytoplasm and regulates nucleocytoplasmic transport through pores. Many details of its formation are still unclear. At fertilization the sperm devoid of nuclear envelope pores enters the egg. Although most of the sperm nuclear envelope disassembles, remnants of the envelope at the acrosomal and centriolar fossae do not and are subsequently incorporated into the newly forming male pronuclear envelope. Remnants are conserved from annelid to mammalian sperm.

**Methodology/Principal Findings:**

Using lipid mass spectrometry and a new application of deuterium solid-state NMR spectroscopy we have characterized the lipid composition and membrane dynamics of the sperm nuclear envelope remnants in isolated sperm nuclei.

**Conclusions/Significance:**

We report nuclear envelope remnants are relatively fluid membranes rich in sterols, devoid of sphingomyelin, and highly enriched in polyphosphoinositides and polyunsaturated phospholipids. The localization of the polybasic effector domain of MARCKS illustrates the non-nuclear aspect of the polyphosphoinositides. Based on their atypical biophysical characteristics and phospholipid composition, we suggest a possible role for nuclear envelope remnants in membrane fusion leading to nuclear envelope assembly.

## Introduction

At fertilization, the sea urchin egg is activated by the sperm whose nucleus enters with an envelope devoid of pores. Most of this envelope is rapidly disassembled, its membranes vesiculating as the sperm chromatin decondenses from its compact conoïd shape to a uniformly euchromatic spherical mass. During this process, remnants of the sperm nuclear envelope at the tip and base of the nucleus, which line two cup-shaped cavities (the acrosomal and centriolar fossae), are not disassembled [1]. Egg membranes bind to this nucleus and then fuse to form a nuclear envelope with pores, incorporating the polar sperm nuclear envelope remnants into the new male pronuclear envelope [1].

Similar remnant membranes are conserved from annelid to mammalian sperm [2], [3]. Their function has mainly been studied in cell free preparations from fertilized sea urchin egg cytoplasm using a well-established system for nuclear envelope assembly on exogenously added sperm [4]–[6]. From these studies three major points have emerged: 1) without the envelope remnants the egg nuclear membrane precursor vesicles do not bind to the nucleus and thus nuclear envelope formation is prevented, 2) the binding of the egg nuclear membrane precursor vesicles proceeds progressively from the sites of the remnants in the acrosomal and centriolar fossae, eventually surrounding the entire surface [4], and 3) one subfraction of egg vesicles (MV1) is required for the remnants to be incorporated into the new nuclear envelope and can fuse with the remnants without the other vesicles [7]. Thus the remnants are likely to have a role both in binding of precursor membranes and initiation of membrane fusion [6].

To mimic *in vivo* envelope disassembly, sperm nuclei were extracted with a 0.1% non-ionic detergent so that the lateral nuclear envelope was removed and the only membranous material left was at the two poles. We refer to these detergent treated sperm nuclei as 0.1% nuclei. It is important to note that the available evidence suggests these detergent-resistant membranous regions are not created by the extraction procedures since they are visible in untreated sperm, and both *in vitro* and *in vivo* studies have shown that the nuclear envelope remnants become incorporated into the newly formed nuclear envelope [1], [4], [8].

Initially the only physical property attributed to these regions was that they are by definition detergent-resistant membranes (DRMs). DRMs have been subject to a plethora of investigations and the current paradigm predicts that DRMs are typically enriched in cholesterol and saturated phospholipids [9]. DRMs are commonly isolated from whole cell lysates by sucrose gradient centrifugation and purified using specific protein markers such as glycosyl-phosphoinositide (GPI)-anchored proteins [10]–[12]. DRMs obtained in this manner are enriched in cholesterol. Cholesterol confers resistance to detergent solubilization and increases phospholipid packing [9], [13].

Cholesterol has several functions in natural membranes, including acting as a scaffold for targeting various proteins [14], [15]. Cholesterol is also a fusogenic lipid [16]. Its fusogenic properties are due to its high spontaneous negative curvature and thus it has been suggested that when localized it can induce membrane fusion like other lipids displaying negative curvature such as diacylglycerol and phosphatidylethanolamine [16]–[20].

Nuclear envelope remnants are essential for formation of the male pronucleus. During this process, a subset of membrane vesicles derived from the egg cortex called MV1, highly enriched in PLCγ and PtdIns(4,5)P_2_ [21], binds exclusively to the nuclear envelope remnant regions, and other membrane vesicles derived from the egg endoplasmic reticulum accumulate along the sides of the nucleus [22]. All of these subsequently fuse to form a nuclear envelope with functional pores [23], [24].

In this paper we characterize the morphology, composition and dynamics of the remnants. We used lipid mass spectrometry to determine lipid composition and deuterium solid-state NMR spectroscopy to study membrane dynamics of the nuclear envelope remnants. We show they consist of two bilayers with an atypical composition of polyphosphoinositides: 12 mol% PtdInsP, 12 mol% PtdInsP_2_, and 9 mol% PtdInsP_3_ of total phospholipids. They are also rich in cholesterol (42 mol% of total lipids), lack sphingomyelin and are virtually devoid of fully saturated phospholipids. Their fluidity is distributed from liquid ordered to fluid states. We attribute their fluid characteristics primarily to the high fraction of cholesterol and phospholipids with polyunsaturated chains and low fraction of lipids with saturated chains. Moreover we show that by decreasing the average levels of cholesterol we affect nuclear envelope formation, indicating a role for high levels of cholesterol in membrane fusion.

Although our group has previously reported that MV1 also has atypically high amounts of phosphoinositides [21], we believe that this is the first time that natural membrane bilayers with both high levels of sterols and polyphosphoinositides and with fluid properties have been reported. Based on the unusual biophysical properties of the nuclear envelope remnants we suggest a possible role for these membranes in nuclear envelope formation.

## Materials and Methods

### Materials

Sea urchins *Lytechinus pictus* (*L.pictus*) and *Strongylocentrotus purpuratus* (*S. purpuratus*) were purchased from Marinus (Long Beach, CA). *Paracentrotus lividus* (*P. lividus*) were provided by the Unidade de Investigaçao de Biologia do Desenvolvimento (UIBD), Universidade Lusófona, Lisbon, Portugal. DiOC_6_ (3′3-dihexyloxacarbocyanine iodide) was from Invitrogen. The internal standards PtdIns(4)P (diC_16_, H^+^), PtdIns(4,5)P_2_ (diC_16_, H^+^), PtdIns(3,4,5)P_3_ (diC_16_, H^+^) were from Cell Signals, DiC_16_ PtdIns (monosodium salt) was from Echelon and 1,2-dilauroyl-sn-glycero-3-phosphocholine (DLPC), 1,2-dilauroyl-sn-glycero-3-phosphoethanolamine (DLPE), 1,2-dilauroyl-sn-glycero-3-[phospho-L-serine] (sodium salt) (DLPS), 1,2-dilauroyl-sn-glycero-3-phosphate (monosodium salt) (DLPA), 1,2-dilauroyl-sn-glycero-3-[phospho-rac-(1-glycerol)] (sodium salt) (DLPG) were from Avanti Polar Lipids, Inc. The deuterium labelled POPC-^2^H_31_ was from Avanti Polar lipids, Inc. Methyl-beta cyclodextrin (MβCD) and filipin III were from Sigma-Aldrich. The Texas Red MARCKS peptide (residues 151-175) has been previously described [25], and was a gift from Stuart McLaughlin.

### Sperm extracts

The 0.1% nuclei were prepared as previously described [26]. Briefly, 250 μl of concentrated viable sperm was suspended in 10ml of ice-cold SXN buffer (50mM HEPES, 250mM sucrose, 150mM NaCl, 300mM glucose, 0.5mM spermidine, 0.15mM spermine, pH 7.2) and centrifuged at 2,600g for 5 min at 4°C. The pellet was resuspended in 1.5ml SXN and sonicated in an Ultrasonic bath for 6 min. Sperm were centrifuged at 2,600g, 4°C for 1.5 min. The pellets were resuspended in 990 μl SXN and 10 μl of 10% Triton X-100 and further mixed for 30 min at room temperature. These nuclei are referred to as 0.1% nuclei. The nuclei were centrifuged at 2,600g for 2 min at 4°C, washed twice with 1ml of SXN buffer at 2,600g, 1 min at 4°C and finally resuspended in 500 μl SXN plus 500 μl freezing buffer. This suspension was thoroughly homogenized, cryogenically frozen in liquid nitrogen and stored at −80°C.

The use of Triton X-100 in this procedure, and potential nuclei extract contamination does not appear to be detrimental to subsequent reactions based on the following observations: 1) 0.1% nuclei are repeatedly washed after Triton extraction (see above). 2) 0.1% and 1% Triton X-100 extracted nuclei both decondense at the same rate in a soluble fertilized egg cytosolic fraction (S150), indicating that nuclear architecture is intact after exposure to Triton [4]. 3) NERs can be removed from 0.1% nuclei with 1% Triton X-100, and subsequently reassembled into the acrosomal and centriolar fossae, indicating that exposure to high Triton concentrations does not alter NER structure and function [2].

### Decondensation assay

The 0.1% nuclei stored at −80°C were thawed on ice, centrifuged for 2 min at 1,500g, 4°C and gently resuspended in 50 μl Tris-saline buffer (TN: 10mM Tris-HCl, 150mM NaCl, pH 7.2) to a concentration of approximately 6×10^7^ nuclei/ml. Decondensation assays typically consisted of adding 3 μl of nuclei to 20 μl of egg cytoplasm (S10) supplemented with 1.2 μl of ATP-generating system (ATP-GS: 33mM ATP, 333mM creatine phosphate and 0.83mg/ml creatine phosphate kinase) in a 1.5ml microcentrifuge tube (final concentration ∼7.4×10^6^ nuclei/ml). This mixture was incubated at room temperature for 1h with agitation every 15 min. By the end of this period, 0.1% nuclei decondensed and egg membrane precursor vesicles (MVs) were bound to the chromatin. Excess MVs were discarded after centrifugation of nuclei through 30 μl of TN containing 0.5M sucrose in a 1.5ml tube at 750g for 15 min at 4°C. The pellet was processed for electron microscopy.

### Nuclear envelope assembly in a cell-free system

0.1% *L. pictus* nuclei were thawed as above and after centrifugation were resuspended in 450 μl SXN buffer, supplemented with 10mM MβCD or vehicle control. Samples were incubated for 30 min at room temperature with periodic agitation followed by centrifugation at 1500g, 2 min, 4°C and resuspension in 50 μl TN buffer. MVs were bound to control nuclei as described in the previous section (untreated), or nuclei treated with 10mM MβCD. After 1h extracts were supplemented with 1mM GTP to induce fusion of MVs, or left unsupplemented as a negative control. After 2 hr, excess MVs were removed as above and nuclei were stained with 0.015mg/ml DiOC_6_ and viewed under a 100× oil immersion objective. DiOC_6_ was excited with the 488nm line of an Argon/Krypton laser, and the resulting fluorescence was separated using a combination of a dichroic beamsplitter (Q495LP; Chroma) and a HQ510/20nm emission filter. Alternatively, decondensed nuclei were observed with phase contrast. Images were captured with a Hamamatsu Orca camera and processed in OpenLab. Twenty nuclei were scored for NE formation on three independent occasions on the basis of their having a continuous fluorescent rim, and the mean and SEM of these results calculated.

### Transmission electron microscopy (TEM)

For TEM, sperm or 0.1% nuclei were washed once in SXN and centrifuged at 500g, 4°C for 10 min or 1500g, 4°C for 2 min respectively. Pellets were fixed for 1 h in 0.1M Sorensen's buffer (81mM Na_2_HPO_4_, 19mM NaH_2_PO_4_ in distilled water, pH 7.4) containing 2.5% (v/v) glutaraldehyde and 1% (w/v) tannic acid. The pellet was washed in Sorensen's buffer by centrifugation at 500g for 10 min at 4°C. Samples were post-fixed in 1% (v/v) osmium tetroxide in 0.05M Sorensen's buffer for 30 min, washed and dehydrated in an ascending ethanol series and embedded in Araldite over 2 days. Thin sections of approximately 80nm were cut and observed on a JEOL 1010 TEM.

### Cryo-TEM of nuclear envelope remnants

The method was adapted from Möbius *et al.* [27]. The 0.1% nuclei were fixed in 4% (v/v) formaldehyde in 0.1M PHEM (60mM PIPES, 25mM HEPES, 2mM MgCl_2_, 10mM EGTA, pH 6.9) for 30 min on ice and infiltrated with 1.7M sucrose containing 4% (v/v) formaldehyde. Droplets containing sperm cells were put on cutting pins and frozen in liquid nitrogen. After cryo-sectioning, sections were picked up and thawed according to Liou *et al.* [28] in a 1∶1 mixture of 2.3M sucrose and 2% (v/v) methylcellulose.

### Colorimetric determination of total phospholipids and cholesterol/cholesteryl ester (sterols)

Phospholipid concentration was determined by indirect measurement of inorganic phosphates liberated from extracted lipids. This method was adapted from Rouser *et al.* [29]. Extracted lipids (see section “Mass spectrometry analysis”) were dissolved in 500 μl of TN buffer and transferred to glass test tubes. 500 μl of TN, water blank and K_2_HPO_4_ standard solutions ranging from 1 μM to 400 μM were transferred to glass test tubes. 100 μl of 10N sulfuric acid were added to each tube and the tubes were heated for 1.5h at 200°C. 100 μl of 72% (v/v) perchloric acid were added to each tube and the tubes heated for 60 min. 2ml of a distilled water/6N sulfuric acid/2.5% ammonium molybdate (w/v)/10% ascorbic acid (w/v) (7∶1∶1∶1 volume ratio) solution were added to the samples and they were incubated at 50°C for 30 min. Absorption was measured at 820nm. Cholesterol and cholesteryl ester concentrations were determined from lipid-extracted samples. Lipid pellets resuspended in 5 μl TN buffer and 5 μl of cholesterol standard solutions ranging from 0.3 to 7.8mM were supplemented with 500 μl of cholesterol liquid stable reagent provided by Thermo Electron Corporation. The solutions were probe-sonicated (Soniprep 150) at power 10 for 3 seconds and incubated for 5 minutes at 37°C. Absorption was measured at 500nm. From these assays the relative amounts of cholesterol to total lipids was determined. In the text, mol% refers to relative amount of cholesterol compared to total lipids including cholesterol.

### Fluorescence imaging of 0.1% nuclei with filipin III

0.1% nuclei were fixed in 4% (v/v) formaldehyde in SXN buffer for 2.5 h at 4°C. Nuclei were centrifuged at 200g for 10 min at 4°C and resuspended in 30 μl of PHEM buffer. Filipin solution was 50 μg/ml in PHEM buffer [30]. 1×10^6^ nuclei were incubated in 1ml of filipin solution. Staining was carried out on a roller at 4°C for 4h. Nuclei were centrifuged at 200g for 10 min at 4°C and washed twice in 500 μl PHEM buffer. Nuclei were resuspended in 60 μl of PHEM buffer and counted using a hemocytometer. Volumes were adjusted to obtain an equivalent concentration of nuclei in all samples. 5 μl of nuclei were supplemented with 45 μl of PHEM buffer containing the anti-fade DABCO (100mg DABCO in 1ml of PHEM buffer). Filipin was excited at 360nm using a mercury source (Nikon Ltd) combined with a DAPI excitation filter (Nikon Ltd). Fluorescently labelled nuclei were viewed on a modified TE 2000 inverted microscope (Nikon Ltd). Images were captured with a Hamamatsu Orca camera and processed in OpenLab.

### Fluoresence labeling of nuclei with filipin III

Unfixed *L. pictus* 0.1% nuclei were either untreated or extracted with 10mM MβCD as above and stained with filipin solution as described in the previous section. After PHEM washes, nuclei were counted and sample volumes adjusted to give an equal concentration of nuclei per sample. 50 μl of sample was transferred to a quartz cuvette and excited at 360nm by an arc lamp at 75W. Emission spectra were obtained from 400–650nm in a QM6/2005 spectrofluorimeter (PTI) using Felix32 Analysis software V1.0. The emission peak at 479/480nm was measured, and values normalized to control reactions. Additionally, the autofluorescence of unstained nuclei was measured and subtracted from filipin stained nuclei.

### Quantification of phospholipids by HPLC coupled to tandem mass spectrometry (HPLC-ESI-MS/MS)

Biological samples were extracted in silanized glassware according to a modified Folch procedure [31]. 200 μl of sample were added to 4ml of acidified chloroform/methanol (2.5∶1). The mixture was probe-sonicated for 10sec. Samples were filtered through a 0.22 μm Durapore membrane and supplemented with 0.2 volumes of 0.2M K_4_EDTA pH 6.8 solution. The extracted samples were centrifuged at 800g for 15 min at 4°C. The organic phase was dried at 37°C under nitrogen. The lipid pellet was resuspended in 100 μl chloroform/methanol/water (5∶5∶1) and transferred to a 150 μl silanized insert. The lipids were dried under nitrogen and supplemented with 2 μg of phospholipid internal standards (DLPC, DLPE, DPPI, DLPA, DLPS, DLPG) and 3 μg of phosphoinositide internal standards (PtdIns(4)P, PtdIns(4,5)P_2_ and PtdIns(3,4,5)P_3_). Before use, lipids were resuspended in chloroform/methanol/water (90∶9.5∶0.5) (phase A). A comparison of un-extracted versus extracted internal standards indicated that the filtering step does not lead to a loss of lipids (data not shown).

Mass spectrometry lipid analysis was carried out on an API 3000 instrument equipped with an ESI source (Sciex/Applied Biosystems). Lipids were separated by HPLC prior to detection using a normal phase Luna silica (2) 3 μm column (Phenomenex). A gradient elution protocol, adapted from Pettitt *et al.* [32], from 100% phase A containing 7mM ethylamine to 70% phase B1 [chloroform/acetonitrile/methanol/water (30∶30∶35∶5) containing 10mM ethylamine] over 20 min, was used to separate the phospholipids (PC, PG, PE, PI, PS and PA). The phosphoinositides were separated using a 100% phase A to 55% phase B2 [choroform/acetonitrile/methanol/water (30∶30∶32∶8) containing 10mM ethylamine] gradient over 20 min. Phospholipids were ionized at 4kV at 300°C. Phosphoinositide ionization was carried out under the same conditions except that the temperature was decreased to 150°C. Phospholipid species were determined using precursor scans: +184m/z for PtdCho, −196m/z for PtdEth, −241m/z for PtdIns, neutral loss of 87m/z for PtdSer and −153m/z for PtdAc. The collision energies were respectively: +52V, −45V, −60V, −35V and −40V. In a typical lipid analysis, precursor scans of phospholipids and multiple ion scans of phosphoinositides were acquired.

### Fluoresence labelling of whole sperm and 0.1% nuclei with MARCKS peptide

Whole sperm were concentrated at 500g for 10 minutes, all centrifugations were carried out at 4°C unless otherwise stated. 250 μl of concentrated sperm were resuspended in 10ml MPSW (454mM NaCl, 9.7mM KCl, 24.9mM MgCl_2_, 9.6mM CaCl_2_, 27.1mM MgSO_4_, 4.4mM NaHCO_3_, 10mM Tris-HCl, pH 8.0) and centrifuged at 2600g for 5 minutes. The pellet was resuspended in 1.5ml Ca^2+^-free artificial seawater (454mM NaCl, 9.7mM KCl, 2.5mM NaHCO_3_, 25mM EGTA, pH 8.0). Alternatively 0.1% nuclei (previously cryogenically frozen) were thawed on ice and centrifuged at 1500g for 2 mins. The pellet was resuspended in 250 μl TN. 75 μl of either whole sperm or 0.1% nuclei sample was diluted in 425 μl Ca^2+^-free artificial sea water and Texas Red MARCKS effector domain peptide added to an end concentration of 120nM. The sample was incubated at room temperature in the dark for 30 minutes. Sperm/0.1% nuclei were collected by centrifugation at 1500g for 2 minutes and washed twice with Ca^2+^-free artificial seawater to remove excess MARCKS peptide. To visualise sperm/0.1% nuclei, 50 μl of sample was applied to a poly-lysine-coated coverslip and left for 5minutes to attach. Washing in Ca^2+^-free artificial seawater removed excess cells/nuclei. Whole sperm and 0.1% nuclei were viewed under a 100× oil immersion objective. Samples were excited with a mercury source and the resulting fluorescence was separated using a combination of a dichroic beamsplitter (D595DCLP; Chroma) and a HQ630/60nm emission filter. Images were captured with a Hamamatsu Orca camera and processed in OpenLab. Note that the MARCKS peptide is advantageous in these studies as polybasic peptides are cell permeant [33] allowing the analysis of live whole sperm to eliminate the possibility of fixation artefacts.

### Deuterium-labelled vesicles for incorporation in natural membranes

This method was developed by Garnier-Lhomme *et al.* [34]. Briefly, 0.1% nuclei were labelled with deuterated POPC small unilamellar vesicles (SUVs) obtained by probe-sonication above the lipid transition temperature for at least 15min using a 3mm microprobe for ultrahigh amplitude. The pulse cycles were a 4 sec sonication with a 6 sec interruption. SUVs were centrifuged at 10,000g for 10min in order to collect any metal residue from the probe that could interfere with NMR measurements. Median vesicle size was determined by dynamic light scattering to be 55nm. 5×10^9^ frozen 0.1% nuclei were thawed on ice and centrifuged at 500g for 10min at 4°C. The pellets were resuspended in a total volume of 1ml of deuterium-depleted SXN buffer. Nuclei were centrifuged a second time as above and resuspended in 1ml of deuterium depleted SXN buffer to remove as much deuterated water as possible. For probe incorporation, a probe-to-nuclei lipid molar ratio of 0.6∶1 was used. The mixture was kept in a water bath at 40°C for 30 min and gently agitated every 10 min to avoid precipitation. The sample was transferred to a 10mm rotor.

### Deuterium solid-state NMR spectroscopy

The ^2^H NMR spectra were acquired at 76.8MHz on a Bruker WB Avance DSX 500 (11.75T) spectrometer equipped with a static triple WB 1H/X/Y probe holding an in-house 10mm coil with 10 turns to increase sensitivity. Spectra were acquired by means of the quadrupolar echo pulse sequence 90°x-τ-90°y-τ-acq [35]. The acquisition parameters for model membranes were as follows: spectral window of 500 kHz, ^π^/_2_ pulse width 6.5 μs, recycle delay 1s and echo delay 30 μs. The number of acquisitions was 15k. Samples were allowed to equilibrate 45 minutes at a given temperature before the NMR signal was acquired. Typical experimental temperatures were 10°C for the initial acquisition, increased and equilibrated at 40°C and decreased to 10°C for the final acquisition. Carbon-deuterium order parameters, S_CD_, were calculated as detailed in [34] according to the equation:
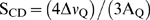
(1)Δν_Q_ is the quadrupolar splitting of the C–D bond considered. In non-oriented spectra, such as those of our study, the only measurable order parameter is that of the so-called “plateau” positions, *i.e.*, labeled carbon positions from C2 to C10 and A_Q_ the static quadrupolar splitting constant: A_Q_ = 167kHz for paraffinic C–D bonds [36]. Model membrane order parameter calculations were from Garnier-Lhomme *et al*. [34].

## Results

### Nuclear envelope remnants line the acrosomal and centriolar fossae of 0.1% nuclei

**Figure 1:**
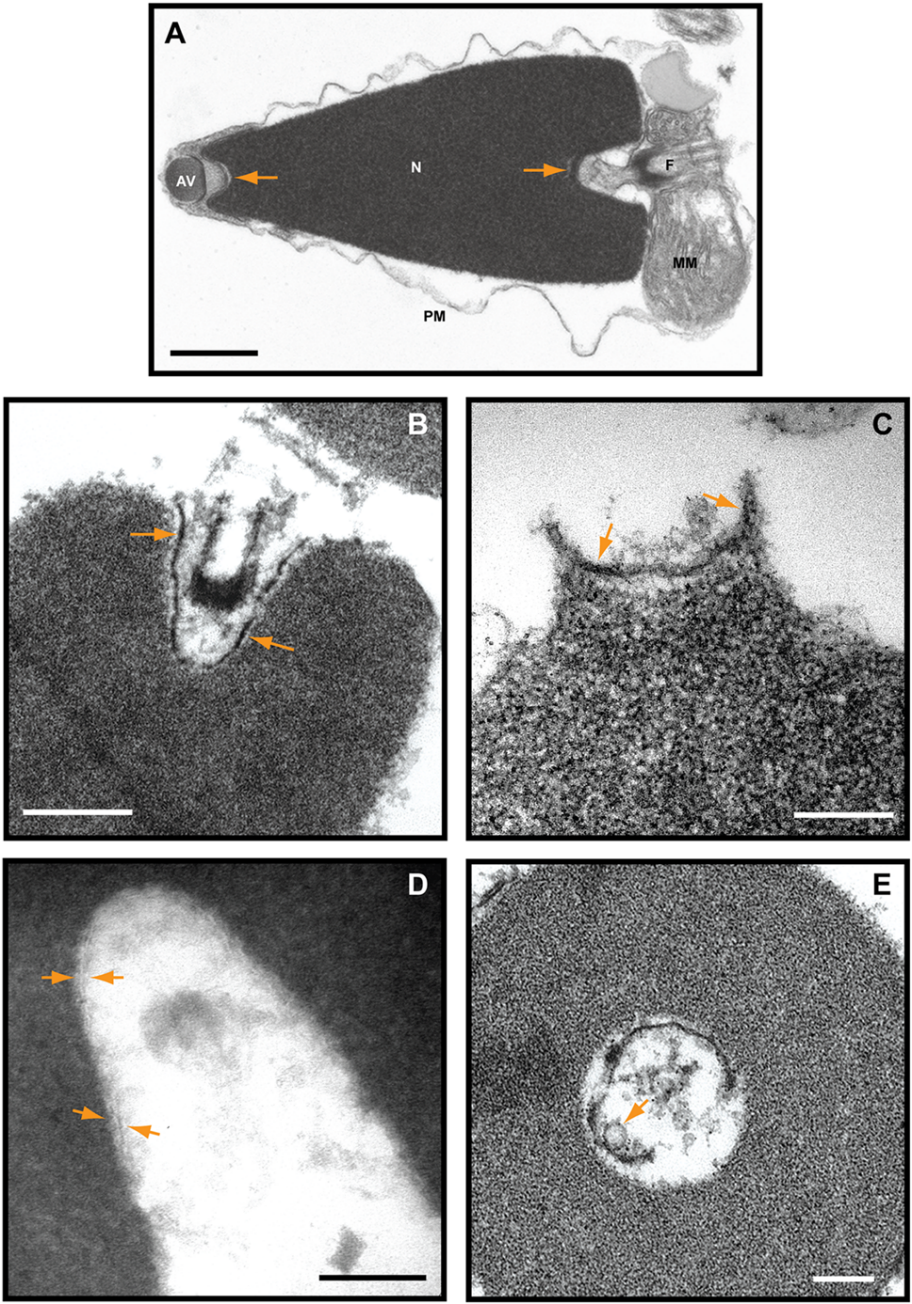
Nuclear envelope remnants contain two membranes that line the acrosomal and centriolar fossae. (A) *P. lividus* sperm cells were fixed in the presence of 1% (w/v) tannic acid. The plasma membrane (PM) and the mitochondrial membranes (MM) are shown. AV: acrosomal vesicle, N: nucleus, F: flagellum. The nuclear envelope is tightly apposed to the chromatin but cup-like structures with nuclear envelope remnants can be seen at the poles (arrows). (B and C) *S. purpuratus* 0.1% nuclei were incubated in egg cytoplasm supplemented with ATP-GS and fixed in the presence of 1% (w/v) tannic acid. Electron dense structures (arrows) are shown in the centriolar (B) and acrosomal fossae (C). The two bilayers appear to have variable amounts of electron dense material between them. (D) Cryosections of *S. purpuratus* 0.1% nuclei prefixed in 4% (v/v) formaldehyde for 3h on ice show two membranes in the centriolar fossa (arrows). (E) *S. purpuratus* 0.1% nuclei were incubated in egg cytoplasm in the presence of an ATP-generating system, fixed in 2.5% (v/v) glutaraldehyde in the presence of 1% (w/v) tannic acid and viewed by TEM. The glancing cross section of the centriolar fossa shows the nuclear envelope remnants and an egg membrane vesicle (arrow) associated with the nuclear envelope remnants. Bars are 500nm (A), 400nm (B) and 200nm (C, D and E). The data are representative of nuclei observed in at least 3 experiments on independent nuclei preparations.

To determine the morphology of nuclear envelope remnants we used transmission electron microscopy (TEM). Figure 1A shows a TEM image of a *P. lividus* sperm cell that was fixed in 2.5% (v/v) glutaraldehyde with 1% (w/v) tannic acid. The plasma membrane (PM) was osmotically swollen during sample preparation. The mitochondrial membranes (MM) and the nuclear envelope (NE) are well resolved. The remnant region is indicated by arrows.

Figures 1B and C show 0.1% nuclei incubated in egg cytoplasm supplemented with ATP-GS and fixed with 1% (w/v) tannic acid present. Electron dense structures of ∼15nm thickness are shown in the centriolar (B) and acrosomal (C) fossae (arrows). Two bilayers are optimally resolved in certain regions where the central electron dense material is not uniform. Each bilayer was ∼6nm thick and may be continuous with each other at the ends. We refer to the structure as a double bilayer. These structures have very similar morphology to nuclear remnant membranes seen *in vivo* after fertilization [1]. Electron dense structures of similar morphology have been particularly well resolved in octopus sperm [37]. To acquire the images of the double bilayer in a less invasive manner, cryo-EM was used. *S. purpuratus* 0.1% nuclei were prefixed in 4% (v/v) paraformaldehyde, cryo-protected in 1.7M sucrose with 4% (v/v) formaldehyde and cryo-sectioned. Double membranes were also resolved and each was ∼7nm thick (arrows in Figure 1D).

*S. purpuratus* 0.1% nuclei were decondensed in egg cytoplasm with the ATP-GS and fixed in 2.5% (v/v) glutaraldehyde for TEM imaging. Figure 1E shows a section where the remnant lines the cupped shaped centriolar cavity with an egg membrane vesicle associated with the envelope remnant for reference (arrow). In places the double bilayer can be observed.

Membrane thickness in mammalian cells ranges from 4 to 7nm depending on the technique used [38]–[41] which agrees closely with our data. When stained with tannic acid, the two bilayers of nuclear envelope remnants were not always resolved clearly, probably due to the tannic acid interacting with either the headgroups of phosphatidylcholine [42] or to material between the parallel bilayers.

### Both phospholipids and cholesterol localize at the nuclear envelope remnants

**Figure 2:**
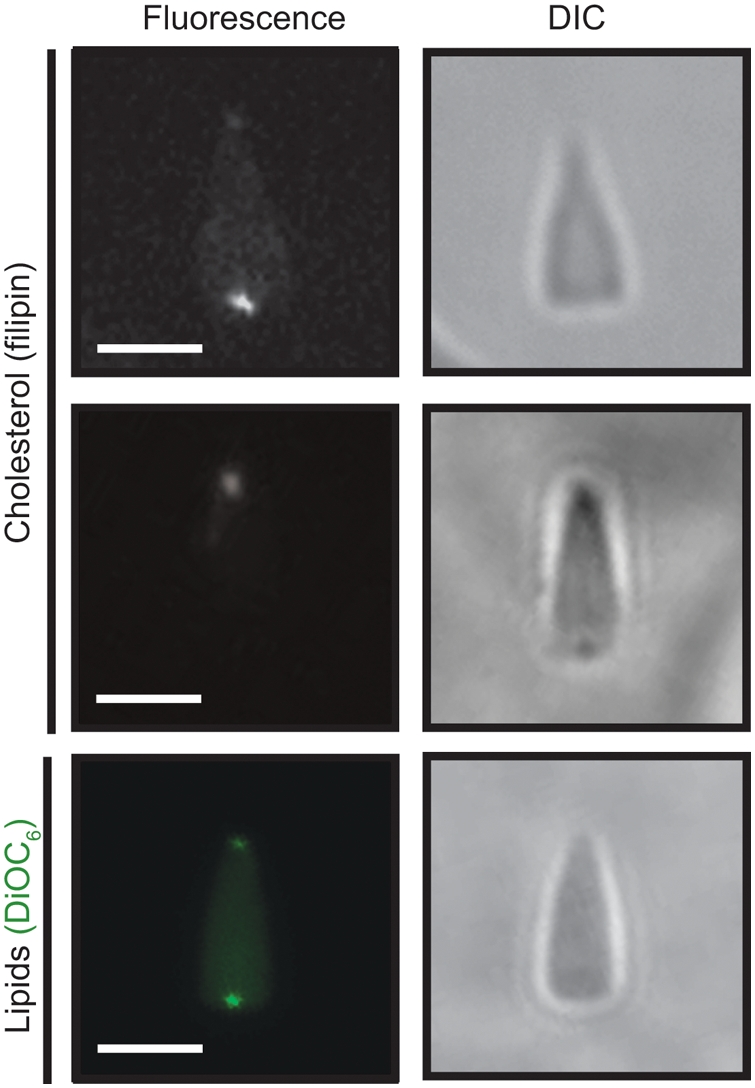
Cholesterol and phospholipids both localize at the poles of the sperm nucleus. The 0.1% nuclei were fixed in 4% (v/v) formaldehyde at 4°C and stained either with filipin to label cholesterol or DiOC_6_ to label nuclear envelope remnants lipids. Cholesterol labelling co-localizes with nuclear envelope remnants at both the acrosomal and centriolar fossae. DIC: differential interference contrast. Bars are 2.5 μm. Note that two panels of filipin staining are shown as in each case only one NER was in the confocal plane. Data are representative of at least 3 experiments performed on independent nuclei preparations.

Given that nuclear envelope remnants are resistant to detergent solubilization, we predicted that they would be enriched in cholesterol. We therefore determined their cholesterol and cholesterol ester (sterol) content by colorimetric assay. Nuclear envelope remnants were 42±10 mol% sterol and 58±10 mol% total phospholipids. The relative amounts of sterols was higher than a typical mammalian plasma membrane of approximately 30 mol% cholesterol [43] but similar to the plasma membrane levels reported for the sea urchins *A. punctulata* (45%) [44] and *L. variegatus* (35%) [45]. To show that the sterols were mainly localized at the nuclear envelope remnants, we labelled 0.1% nuclei with the sterol binding agent filipin. Figure 2 (top and middle left panels) shows two bright spots at both the acrosomal (middle panel) and centriolar fossae (top panel). The sterol signal was not concentrated in the chromatin. To ensure that phospholipids were also localized in the same regions as the sterols, we stained the demembranated nuclei with the lipophilic membrane dye, DiOC_6_ (Figure 2, bottom-left panel). The phospholipids were also located at both fossae, demonstrating that sterols are concentrated at the nuclear envelope remnants, not the chromatin.

### Nuclear envelope remnants are enriched in polyunsaturated phosphoinositides

Once we had determined that the phospholipids and the sterols were at the site of the remnants, we analyzed the detailed phospholipid composition of the nuclear envelope remnants. For this analysis we exploited HPLC coupled to electrospray ionization tandem mass spectrometry (ESI-MS/MS).

The phospholipids of the 0.1% nuclei from *L. pictus* were extracted using a modified Folch method [31]. Phospholipids separated by HPLC on a normal phase column were characterized by ESI-MS/MS using the precursor ion scans of sphingomyelin (SM), phosphatidylglycerol (PtdGly), phosphatidylethanolamine (PtdEth), phosphatidic acid (PtdAc), phosphatidylserine (PtdSer), phosphatidylcholine (PtdCho) and phosphatidylinositol (PtdIns) or using the multiple ion scans of phosphatidylinositolphosphate (PtdInsP), phosphatidylinositolbisphosphate (PtdInsP_2_) and phosphatidylinositoltrisphosphate (PtdInsP_3_). They were quantified relative to a mixture of internal standards.

**Figure 3:**
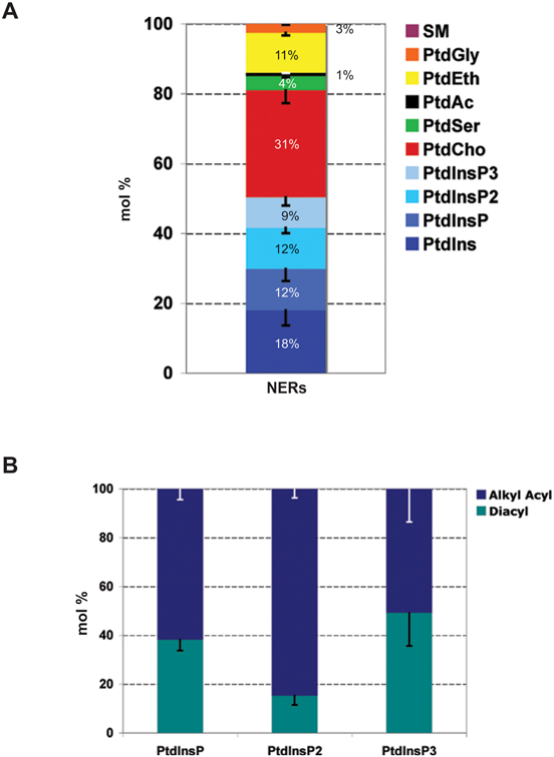
Nuclear envelope remnants are enriched in polyphosphoinositides. Lipid analysis of nuclear envelope remnants. (A) Lipids extracted from *L. pictus* demembranated sperm cells were separated by HPLC on a normal phase column and characterized by ESI-MS/MS using the precursor ion scans of sphingomyelin (SM), phosphatidylglycerol (PtdGly), phosphatidylethanolamine (PtdEth), phosphatidic acid (PtdAc), phosphatidylserine (PtdSer), phosphatidylcholine (PtdCho), phosphatidylinositol (PtdIns) or using the multiple ion scans of phosphatidylinositolphosphate (PtdInsP), phosphatidylinositolbisphosphate (PtdInsP_2_) and phosphatidylinositoltrisphosphate (PtdInsP_3_). Phospholipids were quantified using 12∶0/12∶0 (SM, PtdGly, PtdEth, PtdAc, PtdSer and PtdCho) or 16∶0/16∶0 (PtdIns, PtdInsP, PtdInsP_2_ and PtdInsP_3_) internal standards. Data expressed as mean±SEM (n = 3). (B) Alkyl-acyl versus diacyl phosphoinositides species distribution in nuclear envelope remnants. Mole percentages of diacyl species (green) and alkyl-acyl species (blue) were quantified from the multiple ion scans for each phosphoinositide class: PtdInsP, PtdInsP_2_ and PtdInsP_3_. 38% of PtdInsP, 15% of PtdInsP_2_ and 49% of PtdInsP_3_ are diacyl species. The PtdInsP_2_ is predominantly alkyl-acyl phosphoinositide. Data expressed as mean±SEM (n = 3).

Figure 3A shows the composition of the nuclear envelope remnants. Most striking is the high level of phosphoinositides and complete lack of sphingomyelin. Of total phospholipid content, the nuclear envelope remnants were 18±4 mol% PtdIns, 12±3 mol% PtdInsP, 12±2 mol% PtdInsP_2_ and 9±2 mol% PtdInsP_3_. The level of all PtdIns species together is 51%, much higher than the plasma membrane levels reported for *A. punctulata* (26%) [44] or cytoplasmic membranes in the sea urchin *L. pictus* (27%) [21]. Therefore the remnants have the highest values of total PtdIns species reported for any natural membrane other than MV1 of *L. pictus* (60%) [21].

Quantification of diacyl (Figure 3B-green) and alkyl-acyl (Figure 3B-blue) species from the multiple ion scans of the phosphoinositides showed that 38±4% of PtdInsP, 15±4% of PtdInsP_2_ and 49±14% of PtdInsP_3_ were diacyl lipids. Note that PLCγ prefers diacyl PtdIns(4,5)P_2_ as its substrate [21] and *L. pictus* MV1 vesicles highly enriched PLCγ are 76% diacyl PtdInsP_2_ whereas PtdInsP_2_ of the nuclear envelope remnants is predominantly alkyl-acyl. Also shown in Table 1, the major species of PtdCho, PtdEth and PtdIns were almost all polyunsaturated with the dominant species of each being arachidonyl (20∶4).

**Table 1:** Nuclear envelope remnant phospholipid species are mainly polyunsaturated and arachidonyl.

PtdCho species	PtdEth species	PtdIns species
18∶1/20∶4	18∶0/20∶1	18∶0/20∶4
18∶0/20∶4	18∶0/20∶0	18∶2/20∶4
20∶1/20∶4	18∶1/20∶4	20∶1/20∶4
20∶1/20∶3	18∶0/20∶4	20∶0/20∶4
20∶2/20∶4	18∶0a/20∶4	18∶0a/20∶4
16∶0/20∶4	20∶0a/20∶4	20∶0a/20∶4
20∶1a/20∶4		
18∶0a/20∶4		
16∶0/20∶3		
18∶2/20∶4		
18∶1a/20∶4		
20∶0/22∶6		
20∶1a/20∶5		
20∶0/24∶4		
24∶0/24∶5		
20∶0/24∶5		
24∶0/24∶6		
16∶0a/20∶4		

PtdCho, PtdEth and PtdIns species extracted from *L. pictus* 0.1% nuclei were characterized using the precursor ion scans of +184m/z, −196m/z and −241m/z respectively. Both alkyl-acyl (denoted by ‘a’) and diacyl species were mostly arachidonyl on their sn_2_ positions. Lipid species are listed by descending order of abundance.

**Figure 4:**
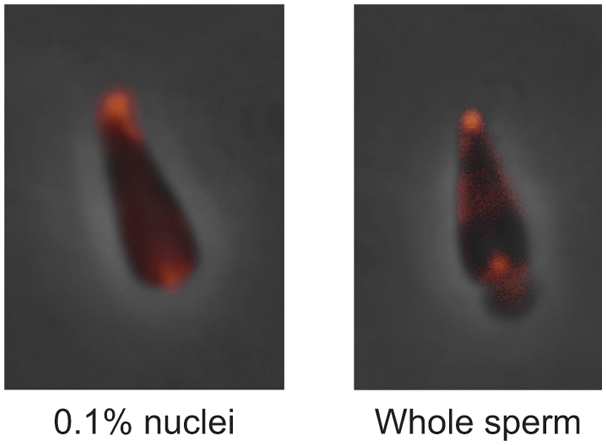
Poly-phosphoinositides in 0.1% nuclei and whole sperm are enriched in the acrosomal and centriolar fossae. *L. pictus* 0.1% nuclei (left) and whole live sperm (right) were incubated with the Texas Red labelled MARCKS peptide and visualised by fluorescence microscopy. The punctate staining of the acrosomal and centriolar fossae is typical of the majority of nuclei observed in experiments on two independent sperm and 0.1% nuclei preparations.

To corroborate our mass spectrometry lipid data we determined the localization of poly-phosphoinositide lipids on 0.1% nuclei. This was undertaken with a Texas Red MARCKS peptide containing a polybasic series of residues that form non-specific electrostatic interactions with all negatively charged phospholipids, but preferentially with the more highly phosphorylated phosphoinositides. The MARCKS peptide will sequester PtdIns(4,5)P_2_ preferentially over PtdSer, even when the latter is 300-fold in excess [25]. Figure 4 shows the probe bound specifically to the regions of 0.1% nuclei corresponding to the NERs. This indicated that the poly-phosphoinositides detected in the ESI-MS/MS lipid analysis were localized in the NERs, and not within the chromatin region. The nuclei of eukaryoates are known to contain pools of phosphoinositides distinct from the lipids of the nuclear envelope [46], [47]. To rule out that the NER phosphoinositide enrichment was not an artifact created by a preferential retention of nuclear matrix phosphoinositides during Triton extraction, we stained live whole sperm with Texas Red MARCKs. The peptide bound preferentially to the region of the NERs in over 80% of stained sperm examined. These data together indicate that NERs are enriched in poly-phosphoinositides in both live whole sperm and 0.1% nuclei. Furthermore, the lipid composition of NERs is not artificially created by detergent extraction.

### Probing microfluidity of nuclear envelope remnants by deuterium solid-state NMR spectroscopy

Given the lipid composition, we next addressed the dynamics of these membranes so high in cholesterol and polyphosphoinositide content. Our initial prediction was that the high levels of cholesterol would cause high molecular order and thus the nuclear envelope remnants would be relatively rigid membranes. To verify this hypothesis we used a method that would directly assess the order parameter of the nuclear envelope remnants.

We previously developed a deuterium solid-state NMR spectroscopy method for direct measurements of membrane dynamics [34]. We used 16∶0(^2^H_31_)/18∶1 PtdCho (POPC-^2^H_31_) small unilamellar vesicles (SUVs) to deliver deuterated phosphatidylcholine and assess the dynamics of the nuclei devoid of lateral membranes but containing nuclear envelope remnants. Briefly, 0.1% nuclei (7×10^8^ nuclei, 0.5 μmol lipids) were incubated with 0.29 μmol POPC-^2^H_31_ SUVs for 30 minutes at 40°C. Parallel experiments were performed with SUVs alone to show that the quadrupolar splitting variations were not due to the fusion of POPC-^2^H_31_ SUVs with each other.

**Figure 5:**
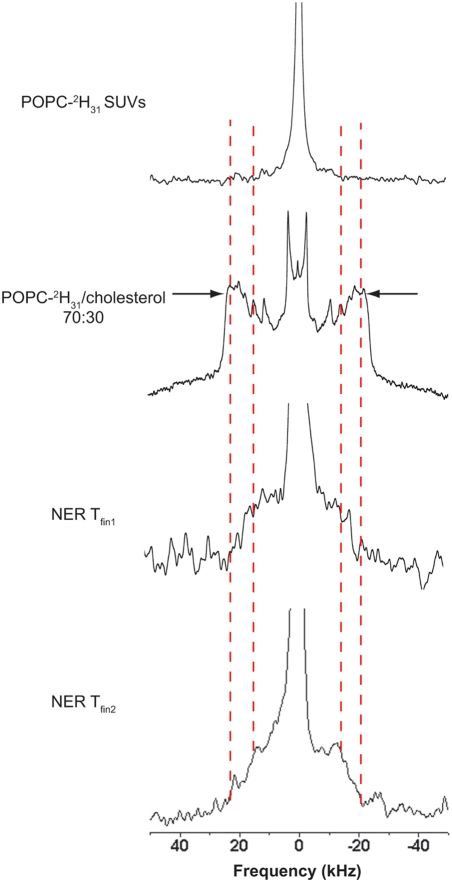
Nuclear envelope remnants are relatively fluid membranes despite their enrichment in cholesterol. The top solid-state deuterium NMR spectrum corresponds to POPC-^2^H_31_ small unilamellar vesicles (SUV) acquired at 10°C post equilibrium at 40°C for 22h. The second spectrum corresponds to POPC-^2^H_31_ MLVs containing 30 mol% cholesterol acquired at 10°C after equilibration at 40°C. The bottom two spectra are of 0.1% nuclei incubated with SUVs of deuterated lipid (POPC-^2^H_31_) for 30 min at 40°C. Labelled nuclear envelope remnants were equilibrated at 40°C for 22h and the ^2^H NMR spectrum was acquired post-equilibrium at 10°C for 4h (NER Tfin_1_) and 20h (NER Tfin_2_). The dashed lines show the plateau quadrupolar splitting enlargement of labelled nuclear envelope remnants post-equilibrium. Arrows show the plateau quadrupolar splittings used to calculate the order parameters. NMR spectra are representative of those obtained in a duplicate experiment.

Figure 5 (top panel) shows the POPC-^2^H_31_ SUV spectrum acquired at 10°C following treatment at 40°C for 22h. The isotropic peak, characteristic of small objects, was unchanged after treatment, demonstrating that the SUVs did not fuse to one another at higher temperatures. The panel below shows the ^2^H solid-state NMR spectrum of POPC-^2^H_31_ multilamellar vesicles (MLV) containing 30 mol% cholesterol acquired at 10°C. The broader quadrupolar splitting is referred to as “plateau” quadrupolar splitting and Δν_Q__plateau_ (arrows of Figure 5) corresponds to the most restricted C–D bonds located close to the lipid glycerol backbone (labelled carbon positions C2 to C10). The Δν_Q__plateau_ of MLVs containing 0 mol%, 15 mol% and 30 mol% cholesterol were respectively 28.9kHz, 35.9kHz and 45.0kHz (Table 2). Such values also indicate that the lipids are in a lamellar phase (40)

**Table 2:** Order parameters (2S_CD_) for deuterium labelled POPC incorporated into nuclear envelope remnants and model membranes (MLVs).

Sample	Cholesterol content in mol%	Δν_Q_	2S_CD_
POPC MLVs with cholesterol [34]	0	28.9	0.458
	5	32.2	0.514
	15	35.9	0.573
	30	45.0	0.718
Nuclear Envelope Remnants	42 ± 10 sterols	42/30	0.67/0.49

Cholesterol/cholesteryl ester and phospholipid concentrations were determined by colorimetry. Cholesterol contents are expressed as molar percentages relative to total phospholipids. Nuclear envelope remnant order parameter was calculated from the ^2^H NMR spectrum of labelled nuclear envelope remnants acquired post-equilibrium (T_fin_). POPC MLVs containing 0, 5, 15 or 30 mol% cholesterol values are taken from Garnier-Lhomme *et al.* [34]. Deuterium spectra of labelled nuclear envelope remnants and MLVs were acquired at 10°C after equilibration at 40°C.

Labelled nuclear envelope remnants were first equilibrated at high temperature (40°C for 22h) and a spectrum acquired post-equilibrium at 10°C for 4h (Figure 5, “NER T_fin1_”). Plateau quadrupolar splittings were not measured in this spectrum due to the low signal-to-noise ratio. To obtain a higher signal to noise ratio, a spectrum was also acquired after 20 hours (Figure 5, “NER T_fin2_”). The isotropic peak was cropped to illustrate the powder pattern and represented about 10–15% of the total area. It was assigned to natural abundance ^2^H_2_O and/or to non-fused SUVs. Comparison of both spectra indicates that spectrum labeled “NER T_fin1_” had not reached equilibrium. The observed spectrum has an unusual shape suggesting a distribution of “plateau” quadrupolar splittings ranging from 42 to 30 kHz (see dashed vertical lines).

The values of the Δν_Q plateau_ of nuclear envelope remnants suggest that the phospholipids were in a lamellar structure. The plateau quadrupolar splittings on the nuclear envelope remnants spectrum post-equilibrium (T_fin2_), were used to calculate the order parameter (2S_CD_). This parameter ranged between 0.67 and 0.49, characteristic of the liquid-ordered phase, as generally promoted in the presence of sterols [48]. The order parameter and the Δν_Q plateau_ of the nuclear envelope remnants were compared to model membranes with variable cholesterol contents treated in the same way (Table 2) [34]. From this data and because the ordering promoted by cholesterol has been shown to be quasi linear with cholesterol concentration in membrane models [49] it can be estimated that the nuclear envelope remnant molecular order would correspond to model membranes with a cholesterol content ranging from 5 to 25 mole%. Since cholesterol esters induce ca. 20% less ordering on membranes [50] the amount of cholesterol esters would be ranging from 6 to 30 mole% if the whole sterol pool was only made of cholesterol esters. To sum up on the observed average ordering, the membrane is more disordered than a model membrane containing more than 40% cholesterol and cholesterol esters.

### Sterol depletion of nuclei inhibits nuclear envelope formation in a cell free system

**Figure 6:**
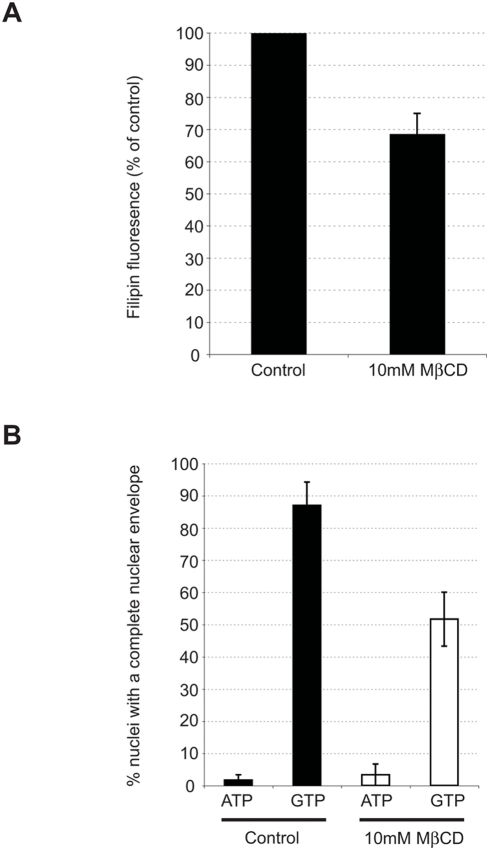
Cholesterol removal from nuclear envelope remnants inhibits membrane fusion events of nuclear envelope formation. (A) 0.1% *L. pictus* nuclei were treated with 10mM MβCD or untreated (control). Nuclei were filipin stained, normalized to an equal nuclei concentration and excited at 360nm. The fluorescence intensity of the emission peak at 479/480nm was measured, and data were normalized to the control value. Data shown are mean±SEM of four experiments conducted in duplicate. (B) 0.1% *L. pictus* nuclei were treated with 10mM MβCD (white) or untreated (black). Nuclear envelope precursor MVs were subsequently bound to nuclei (ATP), and in parallel reactions their fusion was triggered with GTP to complete envelope formation. At least 20 nuclei were scored for the presence of a fully-formed nuclear envelope in 3 independent experiments. Data shown are mean±SEM.

The data presented describe nuclear envelope remnants as double membrane structures with a unique lipid composition that are relatively fluid in nature. The 42 mole% sterol content of nuclear envelope remnants is closest to that of cortical vesicles (57%) [45]. Given the role of cholesterol in facilitating cortical vesicle fusion to the plasma membrane [20] we hypothesized that cholesterol in the nuclear envelope remnants may play a similar role in promoting fusion events of nuclear envelope assembly. To test this, we treated 0.1% nuclei with 10mM MβCD, a sterol depleting agent [51], [52]. Detection of sterols with filipin after MβCD treatment of nuclei indicated that there was considerably less sterols present, as indicated by a decrease of over 30% in filipin fluorescence intensity, compared to untreated nuclei (Figure 6A). Moreover, the same MβCD treated nuclei were less able to support nuclear envelope assembly in a cell free system (Figure 6B). In the presence of a fertilized egg cytoplasmic extract and ATP nuclei decondense to a spherical mass and bind egg MVs which fuse in the presence of GTP to form a nuclear envelope. Binding of MVs to MβCD treated nuclei was not affected but the ability of the MVs to subsequently fuse was inhibited by 35% (Figure 6B). Together, these indicate a functional role for nuclear envelope remnant sterols in the fusion events of nuclear envelope formation.

## Discussion

The nuclear envelope remnants lining the acrosomal and centriolar fossae are natural membranes which have not been created due to artifacts of detergent treatment since they are present in untreated sperm. They reincorporate into the newly forming male pronuclear envelope *in vivo* and *in vitro*, are required for binding of nuclear envelope precursor membranes from the egg and are evolutionarily conserved [1], [2], [4]. We have confirmed by TEM that the nuclear envelope remnants contain two membrane bilayers and demonstrated their lamellar structure by solid-state NMR spectroscopy. Despite their high sterol content, the membranes are more fluid than corresponding models, suggesting that other molecules in NERs counteract the ordering effect produced by sterols.

Although rich in sterols, nuclear envelope remnants are devoid of sphingomyelin, contain very polyunsaturated phospholipids and are strikingly enriched in polyphosphoinositides and arachidonyl phospholipids. Moreover, the fatty acids are mostly saturated on the sn_1_ position and polyunsaturated on the sn_2_ (Table1). Studies on model membranes, where the sn_1_ and sn_2_ are saturated and unsaturated respectively, have shown that cholesterol is intercalated parallel to the phospholipids [53], [54]. Considering the above we speculate that cholesterol function may be to minimize steric and charge repulsions in the bilayer by intercalating between the large polyphosphoinositide headgroups and promote the liquid ordered phase to increase membrane stability. It is of note that Triton X-100 extraction has been proposed to alter the composition of model membranes *in vitro* [55]. We believe this to be highly unlikely for NERs however, as the *in vivo* detection of phosphoinositides with the MARCKS effector domain indicated their enrichment in both live whole sperm and derergent extracted sperm. This is contrary to what one would expect if NERs were a detergent artefact only.

The elevated quantities of polyvalent phospholipids we observe in natural membranes lead to an obvious question of how these lipids might be distributed in the membrane bilayers. At the plasma membrane, lipid distribution is asymmetric with the polyphosphoinositides, present at much lower levels than other phospholipids, and mainly located on the inner leaflet [56]. In the case of nuclear envelope remnants with high levels of polyphosphoinositides, the membrane would be unlikely to be so asymmetric and the phosphoinositides might be distributed on both sides of the bilayer to reduce but not eliminate charge repulsion. Some of the repulsion may be reduced by targeting cytoplasmic PH domain-containing proteins [57] and/or proteins containing basic/hydrophobic clusters such as the MARCKS proteins (Figure 4) and some small GTPases [58], [59] to the PtdInsP_2_ and PtdInsP_3_. The attraction of positive amino acid residues would contribute to neutralizing the highly charged lipids. Likewise, the bilayer of nuclear envelope remnants closer to the chromatin might also have a more symmetric distribution of phosphoinositides with sterols stabilizing the charged lipids and chromatin proteins like histones neutralizing them.

We suggest that the unexpected fluidity of the nuclear envelope remnants results from the dominance of polyunsaturated lipids such as arachidonyl (20∶4) lipids in the phospholipid profile that decrease the ordering effect of high sterol concentration. These could be dispersed on both the cytoplasmic and nucleoplasmic adjacent bilayers. It has been shown that elevated quantities of polyunsaturated PtdIns enhance membrane fluidity [60]. The “detergent resistant” properties of the remnants may be due to the association of the membrane with various proteins or chromatin attachment [37] and to the presence of the liquid-ordered state.

The work reported here leads to a possible explanation for the roles of enriched sterols and polyphosphoinositides in the nuclear envelope remnants. In the sea urchin, the fusion partners of the remnants, vesicles called MV1, are highly enriched in PLCγ and polyphosphoinositides [21], but not sterols [unpublished data]. Indeed they are the only other reported natural membranes containing more than 50% of their phospholipids as phosphoinositides. We have proposed that the role of activated PLCγ in MV1 is to produce from PtdInsP_2_ diacylglycerol, a lipid known to promote negative curvature and hence initiate localized membrane fusion [61]. Indeed, experimentally elevating DAG in MV1 promotes NE formation [61]. In contrast, the nuclear envelope remnants lack PLCγ or its preferred diacyl PtdIns(4,5)P_2_ substrate [unpublished data and [24]]. Instead they are enriched in alkyl-acyl PtdIns(4,5)P_2_ so in the absence of enzyme or substrate, generation of diacylglycerol from PtdIns(4,5)P_2_ in the remnants is unlikely.

Cyclodextrin depletion of sterols to ∼28 mole% typical of most plasma membranes [45] lead to partial inhibition of nuclear envelope formation. We suggest that for nuclear envelope assembly to occur, the sterols in the NERs might be needed to stabilize non-lamellar phases induced by the localized generation of DAG from MV1 to facilitate membrane fusion [62]. The alkyl-acyl PtdIns(4,5)P_2_ in the nuclear envelope remnants, rather than being a source of DAG as in MV1, might instead serve to target proteins with polybasic clusters such as some GTPases which could regulate membrane fusion [58], [59].

In summary the properties and composition of the remnants and MV1 suggests that polyphosphoinositides and sterols, in addition to their many other functions [63], are present in a high mole fraction in some membranes to alter membrane structure so as to facilitate localized fusion. Investigations of the biophysical properties of membranes with similar composition to nuclear envelope remnants should give more direct insight into the roles of these lipids and their involvement in the molecular mechanisms of membrane fusion.
